# Field safety and effectiveness of new visceral leishmaniasis treatment regimens within public health facilities in Bihar, India

**DOI:** 10.1371/journal.pntd.0006830

**Published:** 2018-10-22

**Authors:** Vishal Goyal, Raman Mahajan, Krishna Pandey, Shambhu Nath Singh, Ravi Shankar Singh, Nathalie Strub-Wourgaft, Fabiana Alves, Vidya Nand Rabi Das, Roshan Kamal Topno, Bhawna Sharma, Manica Balasegaram, Caryn Bern, Allen Hightower, Suman Rijal, Sally Ellis, Temmy Sunyoto, Sakib Burza, Nines Lima, Pradeep Das, Jorge Alvar

**Affiliations:** 1 Drugs for Neglected Diseases *initiative* (DND*i*), New Delhi, India; 2 Médecins Sans Frontières (MSF), New Delhi, India; 3 Division of Clinical Medicine, Rajendra Memorial Research Institute of Medical Sciences (RMRI), New Delhi, India; 4 Sadar Hospital Chapra, Saran, India; 5 Drugs for Neglected Diseases *initiative* (DND*i*), Geneva, Switzerland; 6 Department of Epidemiology & Biostatistics, University of California San Francisco, San Francisco CA, United States of America; 7 Independent consultant, Bangkok, Thailand; 8 Médecins Sans Frontières (MSF), Barcelona, Spain; Universidade do Estado do Rio de Janeiro, BRAZIL

## Abstract

**Background:**

In 2010, WHO recommended the use of new short-course treatment regimens in kala-azar elimination efforts for the Indian subcontinent. Although phase 3 studies have shown excellent results, there remains a lack of evidence on a wider treatment population and the safety and effectiveness of these regimens under field conditions.

**Methods:**

This was an open label, prospective, non-randomized, non-comparative, multi-centric trial conducted within public health facilities in two highly endemic districts and a specialist referral centre in Bihar, India. Three treatment regimens were tested: single dose AmBisome (SDA), concomitant miltefosine and paromomycin (Milt+PM), and concomitant AmBisome and miltefosine (AmB+Milt). Patients with complicated disease or significant co-morbidities were treated in the SDA arm. Sample sizes were set at a minimum of 300 per arm, taking into account inter-site variation and an estimated failure risk of 5% with 5% precision. Outcomes of drug effectiveness and safety were measured at 6 months. The trial was prospectively registered with the Clinical Trials Registry India: CTRI/2012/08/002891.

**Results:**

Out of 1,761 patients recruited, 50.6% (n = 891) received SDA, 20.3% (n = 358) AmB+Milt and 29.1% (n = 512) Milt+PM. In the ITT analysis, the final cure rates were SDA 91.4% (95% CI 89.3–93.1), AmB+Milt 88.8% (95% CI 85.1–91.9) and Milt+PM 96.9% (95% CI 95.0–98.2). In the complete case analysis, cure rates were SDA 95.5% (95% CI 93.9–96.8), AmB+Milt 95.5% (95% CI 92.7–97.5) and Milt+PM 99.6% (95% CI 98.6–99.9). All three regimens were safe, with 5 severe adverse events in the SDA arm, two of which were considered to be drug related.

**Conclusion:**

All regimens showed acceptable outcomes and safety profiles in a range of patients under field conditions. Phase IV field-based studies, although extremely rare for neglected tropical diseases, are good practice and an important step in validating the results of more restrictive hospital-based studies before widespread implementation, and in this case contributed to national level policy change in India.

**Trial registration:**

Clinical trial is registered at Clinical trial registry of India (CTRI/2012/08/002891, Registered on 16/08/2012, Trial Registered Prospectively).

## Author summary

Treatment is one of key strategies for visceral leishmaniasis control and elimination. Historically a number of monotherapy drugs for VL treatment were used in India including pentavalent antimonials, amphotericin B deoxycholate (AmB), and miltefosine (MF). With the limited number of drugs available there was a need to preserve existing drugs and to develop shorter and safer treatment regimens. Three short-course combination regimen including AmBisome, miltefosine and paromomycin have been evaluated in a phase III clinical trial conducted in India (2008–2010). All showed an excellent safety profile and an efficacy of at least 97% in controlled conditions. In 2010, WHO recommended the use of new short-course treatment regimens in kala-azar elimination efforts for the Indian subcontinent. Although phase 3 studies have shown excellent results, there remains a lack of evidence on a wider treatment population and the safety and effectiveness of these regimens under field conditions within national program settings. This study was implemented in field conditions with treatment provided by government doctors, providing further evidence for scaling up new regimens in national program contexts within the public health sector and contributing to national policy change in India.

## Introduction

Visceral leishmaniasis (VL, also known as kala-azar) is an ultimately fatal disease with 10,311 reported cases in the Indian subcontinent in 2014 [1], although under-reporting means that the real number is likely to be higher [2]. The number of reported cases in India has progressively declined in recent years from 33,187 in 2011 to 6245 in 2016, an approximate annual reduction of 30–35% [3]; this may be due to a number of factors, including the VL elimination initiative in South-East Asia, the natural incidence cycles of the disease, and improvements in social conditions.

Early and effective treatment is one of the pillars of the VL elimination strategy. Historically, a number of drugs have been used in India in monotherapy, including pentavalent antimonials, amphotericin B deoxycholate, miltefosine, paromomycin, and liposomal or lipid formulations of amphotericin B [4,5]. Pentavalent antimonials, the only available treatment for VL for decades, are no longer recommended in the most endemic state of Bihar due to development of resistance, with treatment failure reaching more than 60% in some villages [6].

Miltefosine was introduced into the national program as an orally administered 28-day monotherapy in 2005, with very satisfactory cure rates. However, its efficacy decreased from 96% to 90% within a decade of use in India [7,8], with higher reported failure rates in children, likely to be related to the inappropriate linear dosage which was used [9].

In Nepal, a 10% failure rate for miltefosine at 6 months doubled to 20% at 12 months follow-up. With limited drugs available, there was a need to preserve the existing drugs and to develop shorter and safer treatment regimens [10]. Amphotericin B deoxycholate is a highly efficacious drug with a cure rate of 97%, but requires in-patient treatment for up to a month, which, coupled with infusion and drug-related adverse effects, has limited its utility [11].

AmBisome (Gilead Pharmaceuticals, Foster City, CA, USA) is a brand name for liposomal amphotericin B (AmB). It has been studied extensively at a range of doses and shows excellent safety and efficacy. In a study carried out by Sundar *et al*., a single 10 mg/kg dose of AmB had 95.7% efficacy and was safer than conventional amphotericin B deoxycholate [11].

An earlier phase III non-inferiority clinical trial in India comparing conventional amphotericin B deoxycholate with three different low-dose combinations (AmBisome 5 mg/kg plus 7 days of miltefosine; AmBisome 5 mg/kg plus 10 days of paromomycin; miltefosine plus paromomycin both for 10 days) found all three to be non-inferior with final cure rates of ≥97% at 6 months [12]. In 2010, the WHO recommended these combination regimens along with a single dose of 10mg/kg AmBisome (known as Single Dose AmBisome/SDA) as first line treatments in South Asia [13] based on economic, safety, and efficacy considerations [14–16].

However, these hospital-based studies were restricted in sample size, conducted under very controlled conditions, and mostly excluded unwell or patients from more vulnerable groups (e.g. pregnant women or the very young/old). As such, the Drugs for Neglected Diseases *initiative* (DND*i*), in collaboration with Rajendra Memorial Research Institute of Medical Science (RMRIMS), State Health Society Bihar, and Médecins Sans Frontières (MSF), conducted this field effectiveness study to better determine the safety and feasibility of these treatment regimens under field conditions within public healthcare facilities in Bihar, India.

## Methods

### Ethics statement

The protocol was approved by the Institutional Ethics Committee of RMRI Patna, Ethics Review Board of Mèdecins Sans Frontières, London School of Hygiene & Tropical Medicine (Ref 6046), Indian Council of Medical Research, Drug Controller General of India and National Vector Borne Disease Control Programme. Written informed consent was obtained by a treating physician. For children, consent of parents or of a legal representative was obtained.

### Trial design

This study was an open label, prospective, non-randomized, non-comparative multicenter phase IV clinical trial conducted through government hospitals and primary health clinics (PHCs) in Bihar state, India. The study was conducted from August 2012 to September 2015 in two districts (Vaishali and Saran) and at the Rajendra Memorial Research Institute of Medical Sciences (RMRIMS), a government research institute specializing in VL located in Patna.

### Inclusion and exclusion criteria

All patients meeting a case definition of VL defined as fever for more than 2 weeks, splenomegaly, and confirmed with a positive rK-39 rapid diagnostic test (InBios, USA) were included in the study. Relapse cases with a confirmatory parasitological diagnosis were also eligible. Patients with concurrent PKDL, HIV and those reporting a history of hypersensitivity to the investigational drugs were excluded.

Upon confirmation of VL, written informed consent was obtained by a treating physician. For children, consent of parents or of a legal representative was obtained. Prior to treatment, blood was taken for haemoglobin, alanine aminotransferase (ALT), aspartate aminostransferase (AST), and serum creatinine. Other tests were performed when medically indicated. For women aged 12–55 years, a urinary pregnancy test was also conducted, with all pregnant women being referred for SDA treatment. Due to the teratogenicity of miltefosine, women with child-bearing potential unwilling to use long-acting injectable contraception during and for three months after treatment were also referred for SDA treatment.

Height and weight were measured for all patients at admission. Anthropometric indicators appropriate for patient age were calculated using the latest World Health Organization (WHO) Multicentre Growth Reference [17]. Severe wasting was defined based on WHO criteria (weight for height Z-score <-3 for children <5 years; BMI-for-age Z-score <-3 for those 5–19 years; and BMI <16.0 for adults). Severe anaemia was defined as haemoglobin <7 g/dL for children <5 years; <8 g/dL for 5 years and older; moderate anaemia defined as <11 g/dL but above the cut-off for severe anaemia [18].

Patients with haemoglobin <4 g/dl, serious concomitant infection (e.g. severe pneumonia), complicated severe malnutrition, TB/VL co-infection, or children <2 years of age were referred to the MSF VL treatment unit within Hajipur district hospital or RMRIMS for further specialist management. These patients were treated with SDA as per physician decision and included in the study.

### Treatments

The three regimens evaluated were: a 10 mg/kg single intravenous dose of AmBisome (SDA); a 5 mg/kg single intravenous dose of AmBisome plus 7 days of linear dosage oral miltefosine (AmB+Milt); and 11 mg/kg intramuscular base paromomycin plus linear dosage oral miltefosine for 10 days (Milt+PM). Linear dosage of miltefosine was 2 doses of 50 mg (morning and evening) for patients ≥12 years weighing more than 25 kg, or a single morning dose of 50 mg for those weighing less than 25 kg. Children of 2–11 years were given miltefosine at a dose of 2.5 mg/kg/day orally divided into two daily doses.

The SDA regimen was administered in 5% dextrose over approximately 2 hours after completion of a test dose of 1 mg to check for hypersensitivity over 30 minutes; patients were discharged the following day from the district hospital where clinical conditions allowed a safe return home. The AmB+Milt regimen consisted of AmBisome 5 mg/kg, administered as above on day 1, with oral miltefosine on days 2 to 8 to be taken at home with advice to return in case of any adverse event. The Milt+PM regimen consisted of the oral miltefosine dose plus intramuscular paromomycin (11 mg/kg/day in a single daily dose) given concomitantly daily for 10 days. Patients treated at the district hospital were admitted to a VL ward for the 10 days of treatment, whereas patients enrolled at PHC level were managed on an outpatient basis, returning each day for the injection. Patients who failed to return for ambulatory treatment were actively traced by telephone and, if necessary, in person to ensure maximum compliance.

Following national regulatory recommendation as part of the study approval process, children were only treated at the district hospitals under the supervision of a paediatrician. At the specialist RMRI facility, all three modalities were used, based on clinician decision. Patients that relapsed with any of the three treatment regimens were given rescue treatment as per physician decision.

### Follow-Up

Patients were asked to return for two post-treatment follow-up visits. The first was scheduled 7–20 days after treatment onset to assess initial cure. A second follow-up visit was planned at 6 months (with a 5–10 month window period) after treatment onset, to assess final cure. Patients were actively traced if they did not attend follow-up visits.

### Outcomes

Treatment stopped was defined as treatment stopped early by the attending clinician for any reason.

Default was defined as failure to finish treatment against medical advice.

Relapse was defined as recurrence of clinical symptoms and visualization of parasites in spleen or bone marrow aspirate before the 6 month follow up period.

Death was reported if it occurred from any cause up to 6-months post-treatment.

Lost to follow-up was defined as a patient who was unable to be traced at the 6 months follow up window.

For effectiveness analyses, the primary outcome was final cure defined as a negative test of cure at the end of treatment, absence of clinical signs and symptoms of VL and no relapse up to 6 months follow-up.

### Data analysis and statistical methods

#### Sample size

Since the objective of the study was to evaluate the effectiveness and safety of each new treatment modality, the sample size requirement was based around the precision with which effectiveness and safety could be estimated. Assuming a risk of failure of 5% at 6-months follow-up, a sample size of 225 patients per arm would allow for an effectiveness estimation with 3% precision. Since treatment modality allocation was planned to be different between sites, and the patient population might not be homogeneous (referral hospital vs PHC in different districts), an adjustment was applied using a conservative design effect of 4 to account for between-centre variability. In this case, a failure risk of 5% could be estimated at around 5% precision with 300 patients per arm.

#### Statistical analysis

Two effectiveness analyses were performed. In the intention-to-treat (ITT) analysis, all patients who received at least one drug dose were included; those with treatment stopped, treatment default, or lost to follow-up at 6 months were considered as treatment failures. In the complete case analysis, those with treatment stopped, default, or lost to follow-up at 6 months were excluded. A single patient with post-kala-azar dermal leishmaniasis (PKDL) treated before the 6-month follow-up was considered a treatment failure for the ITT analysis, but was excluded from the complete case analysis. Analyses were conducted in SAS 9.3 (SAS Institute, Cary, NC, USA). Statistical differences were tested in univariate analyses using Chi Square test, Fisher Exact test, Wilcoxon Rank Sum, or Kruskal-Wallis tests as appropriate. Multivariable logistic regression models were constructed, and model fit tested using the Hosmer and Lemeshow Goodness-of-Fit Test. Inclusion of candidate variables was based on measures of clinical history and severity that were judged to potentially influence disease response; no automated variable selection was used for inclusion. Drug regimen variables were maintained in the model; other variables were eliminated at p>0.05 using a stepwise backwards elimination procedure, in order to construct a model for treatment failure that explored the role of potential risk factors and confounders. Confounding was ruled out for all covariates tested (sex, liver and renal function tests, wasting, severe anemia).

#### Safety

The adverse events reporting period for this trial lasted from the administration of the first dose of study medication until the initial outcome assessment. All adverse events (related or not related to medication) that occurred during the adverse event reporting period specified in the protocol were evaluated by a physician and reported in the register. Each adverse event was classified by the investigator as serious or non-serious. An adverse event was defined as serious if it is either fatal or life-threatening, or requires or prolong hospitalization, or resulting in persistent or significant disability or a congenital anomaly/birth defect. Serious adverse events were recorded from screening until 6-months follow-up and classified by severity, seriousness, relationship to study drug, and resolution.

## Results

A total of 1,761 patients were recruited, 534 (30.3%) children (≤12 years) and 1,227 (69.7%) adults (>12 years). Male predominance was more marked for adults than for children (769/1227 [62.7%] vs 299/534 [56.0%]; p = 0.008). 891 (50.6%) of patients were treated with the SDA regimen, 358 (20.3%) patients were treated with the AmB+Milt regimen and 512 (29.1%) patients were treated with the Milt+PM regimen in the study (Table 1). Milt+PM was used predominantly in Chapra district hospital (Saran District) and Saran PHCs; AmB+Milt in Hajipur District Hospital (Vaishali District) and Vaishali PHCs and SDA almost exclusively in Hajipur District Hospital (Vaishali District). Chapra district hospital treated 378 patients (21.5%), Vaishali district hospital treated 1,052 patients (59.7%), 96 patients (5.5%) were treated in a tertiary referral centre (RMRI), and 235 patients (13.3%) were treated at primary health care centres in both districts (120 in Saran PHCs and 115 in Vaishali PHCs) (S1 Table). Specific regimens were assigned by treatment site, leading to collinearity between regimen and site. (Table 1 and S1 Table).

**Table 1:** Baseline patient characteristics and completeness of follow-up by treatment arm.

	SDA^1^ (N = 891)	AmB+Milt^2^ (N = 358)	Milt+PM^3^ (N = 512)
**Demographic characteristics**			
Mean age (years [SD])	24.8 (16.9)	30.4 (17.6)	23.1 (17.8)
Age range (years)	2–80	3–75	2–70
Age ≤ 12 years N (%)	271 (30.4)	74 (20.7)	189 (36.9)
Age > 12 years N (%)	620 (69.6)	284 (79.3)	323 (63.1)
Male N (%)	510 (57.2)	247 (69.0)	311 (60.7)
			
**Recruitment site**			
Chapra District Hospital (Saran)	4 (0.5)	0	374 (73.1)
Hajipur District Hospital (Vaishali)	828 (92.9)	218 (60.9)	6 (1.2)
RMRI (Patna)	59 (6.6)	25 (7.0)	12 (2.3)
Saran district PHCs	0	0	120 (23.4)
Vaishali PHCs	0	115 (32.1)	0
			
**Clinical characteristics**			
**Weeks of illness**			
Mean [SD]	7.3 (8.4)	6.7 (6.2)	7.0 (6.0)
Median [IQR]	4 (3–8)	4(3–8)	4 (4–8)
**Severe wasting N (%)**^**4**^	143 (16.0)	39 (10.9)	88 (17.2)
**Weight (mean [SD] in kg)**	36.6 (15.2)	40.9 (14.0)	34.6 (15.5)
**Hemoglobin (mean [SD] in g/dL)**	8.5 (2.0)	9.1 (2.0)	9.3 (1.8)
**Anemia**^**5**^			
Mild or none	103 (11.6)	66 (18.4)	91 (17.8)
Moderate	427 (47.9)	189 (52.8)	307 (60.0)
Severe	361 (40.5)	103 (28.8)	114 (22.3)
**Creatinine (mean [SD] in μmol/L)**	0.7 (0.3)	0.8 (0.3)	0.8 (0.3)
**Alanine aminotransferase**			
Moderate elevation (49–199) N (%)	251 (28.2)	128 (35.8)	152 (29.7)
Marked elevation (≥200) N (%)	32 (3.6)	17 (4.8)	11 (2.2)
**Aspartate aminotransferase**			
Moderate elevation (49–199) N (%)	444 (49.8)	189 (52.8)	220 (43.0)
Marked elevation (≥200) N (%)	90 (10.1)	44 (12.3)	33 (6.5)
			
**Completeness of follow-up**			
Initial follow-up N (%)	885 (99.3)	355 (99.2)	508 (99.2)
6-month follow-up N (%)	853 (95.7)	333 (93.0)	498 (97.3)
Time until 6-month follow-up Median (IQR)	195 (191–209)	194 (190–206)	203 (190–238)

^1^Single dose AmBisome

^2^AmBisome + miltefosine

^3^Miltefosine + paromomycin

^4^Severe wasting defined as weight-for-height Z-score <-3 for children <5 years; BMI-for-age Z-score < -3 for those 5–19 years; and BMI <16.0 for adults

^5^Severe anemia defined as hemoglobin <7 g/dL for children < 5 years; <8 g/dL for 5 years and older; moderate anemia defined as falling above the cutoff for severe anemia and <11 g/dL.

Although not significantly consistent, patients treated with SDA and Milt+PM (a majority of whom were treated at district hospitals) were younger, more likely to be female, and to present with severe wasting than those treated with AmB+Milt. The minority of patients treated at the RMRIMS had a significantly longer reported duration of illness (median of 8 weeks as compared to 4 weeks in other sites).

Severe anaemia was more common in the SDA treatment arm. ALT levels were higher in the AmB+Milt arm, whereas AST levels were higher in the SDA and AmB+Milt arms than in the Milt+PM arm (Table 1).

Overall, 1,684 patients (95.6%) completed the 6-month follow-up visit. Thirteen (0.7%) patients did not complete treatment or had their treatment stopped by a study physician, and 64 patients (3.6%) were lost to follow-up at 6 months. Baseline characteristics of patients lost to follow-up at 6 months (n = 64) differed from those who returned (n = 1697) for their follow-up visits (S2 Table).

### 6-month effectiveness analysis

In the ITT analysis, the final cure rate for SDA was 91.4% (95% CI 89.3–93.1), AmB+Milt 88.8% (95% CI 85.1–91.9), and Milt+PM 96.9% (95% CI 95.0–98.2). In the complete case analysis, cure rates were SDA 95.5% (95% CI 93.9–96.8), AmB+Milt 95.5% (95% CI 92.7–97.5) and Milt+PM 99.6% (95% CI 98.6–99.9) (Table 2).

**Table 2:** Cure at 6 months, by treatment regimen and age group.

	SDA^1^	AmB+Milt^2^	Milt+PM^3^
**All ages**			
**Intention-to-treat (N = 1761 patients)**			
Cured^4^ / total	814/891	318/358	496/512
Cure rate % (95% CI)	91.4 (89.3–93.1)	88.8 (85.1–91.9)	96.9 (95.0–98.2)
**Complete case (N = 1683)**^**5**^			
Cured / total	814/852	318/333	496/498
Cure rate % (95% CI)	95.5 (93.9–96.8)	95.5 (92.7–97.5)	99.6 (98.6–99.9)
			
**Age ≤12 years**	** **	** **	** **
**Intention-to-treat (N = 534 patients)**			
Cured / total	250/271	67/74	184/189
Cure rate % (95% CI)	92.3 (88.4–95.1)	90.5 (81.5–96.1)	97.4 (93.9–99.1)
**Complete case (N = 527)**^**6**^			
Cured / total	250/268	67/73	184/186
Cure rate % (95% CI)	93.3 (89.6–96.0)	91.8 (83.0–96.9)	98.9 (96.2–99.9)
			
**Age >12 years**			
**Intention-to-treat (N = 1227)**			
Cured / total	564/620	251/284	312/323
Cure rate % (95% CI)	91.0 (88.4–93.1)	88.4 (84.1–91.9)	96.6 (94.0–98.3)
**Complete case (N = 1156)**^**7**^			
Cured / total	564/584	251/260	312/312
Cure rate % (95% CI)	96.6 (94.8–97.9)	96.5 (93.5–98.4)	100 (98.8–100)

^1^Single dose AmBisome

^2^AmBisome + miltefosine

^3^Miltefosine + paromomycin

^4^Cured defined as initial cure and no VL relapse at 6 month follow-up; treatment interruption, default, loss to follow-up and one patient treated for PKDL 2 months after VL treatment considered as treatment failures in the intention-to-treat analysis.

^5^Excludes 13 patients with treatment interruption or default, one patient treated for PKDL 2 months after VL treatment and 64 patients lost to follow-up

^6^Excludes 1 patient with treatment interruption or default and 6 patients lost to follow-up

^7^Excludes 12 patients with treatment interruption or default, one patient treated for PKDL 2 months after VL treatment and 58 patients lost to follow-up

### Factors associated with relapse at 6 months

Relapse rates varied by drug regimen and were higher for children than for those older than 12 years (Tables 3 and 4). Those with illness that had lasted 8 weeks or less were also more likely to have relapse at 6 months.

**Table 3:** Univariate analyses of factors associated with VL relapse by 6 months, complete case population (N = 1683)^1^.

Factor	Relapse (N = 55)	No relapse (N = 1628)	Odds ratio	p value
** **	n (row %)	n (row %)	(95% CI)	** **
**Regimen**				
SDA^2^	38 (4.5)	814 (95.5)	Referent	
AmB+Milt^3^	15 (4.5)	318 (95.5)	1.01 (0.55, 1.86)	0.974
Milt+PM^4^	2 (0.4)	496 (99.6)	0.09 (0.02, 0.36)	0.0008
**Sex**				
Male	36 (3.6)	977 (96.4)	1.26 (0.72, 2.22)	0.418
Female	19 (2.8)	651 (97.2)	Referent	
**Age**				
2–12 years	26 (4.9)	501 (95.1)	2.02 (1.18, 3.46)	0.011
>12 years	29 (2.5)	1127 (97.5)	Referent	
**Reported length of illness**				
< = 8 weeks	52 (3.9)	1277 (96.1)	4.76 (1.48, 15.35)	0.0089
>8 weeks	3 (0.9)	351 (99.1)	Referent	
**Severe anemia**^**5**^				
Yes	15 (2.7)	535 (97.3)	0.77 (0.42, 1.40)	0.39
No	40 (3.5)	1093 (96.5)	Referent	
**Severe wasting**^**6**^				
Yes	13 (5.0)	249 (95.0)	1.71 (0.91, 3.24)	0.097
No	42 (3.0)	1379 (97.0)	Referent	
**ALT** **≥200**				
Yes	1 (1.7)	59 (98.3)	0.49 (0.07, 3.62)	0.487
No	54 (3.3)	1569 (96.7)	Referent	
**AST** **≥200**				
Yes	4 (2.5)	158 (97.5)	0.73 (0.26, 2.05)	0.550
No	51 (3.4)	1470 (96.6)	Referent	
**Creatinine** **≥1.5**				
Yes	1 (2.5)	39 (97.5)	0.76 (0.10, 5.59)	0.783
No	54 (3.3)	1589 (96.7)	Referent	
**Patient category**				
Primary kala-azar	50 (3.1)	1540 (96.9)	Referent	
Previously treated kala-azar	3 (4.6)	62 (95.4)	1.49 (0.45, 4.91)	0.512
Transferred	2 (7.1)	26 (92.9)	2.37 (0.55, 10.26)	0.249

^1^Excludes 13 patients with treatment interruption or default, one patient treated for PKDL 2 months after VL treatment and 64 patients lost to follow-up at 6 months

^2^Single dose AmBisome

^3^AmBisome + miltefosine

^4^Miltefosine + paromomycin

^5^Severe anemia defined as hemoglobin <7 g/dL for children < 5 years; <8 g/dL for 5 years and older; moderate anemia defined as falling above the cutoff for severe anemia and <11 g/dL.

^6^Severe wasting defined as weight-for-height Z-score <-3 for children <5 years; BMI-for-age Z-score < -3 for those 5–19 years; and BMI <16.0 for adults

**Table 4:** Multivariable logistic regression model of factors associated with VL relapse by 6 months, complete case population (N = 1683)^1^.

Factor	Adjusted Odds ratio	p value
	(95% CI)	** **
**Regimen**		
SDA^2^	Referent	
AmB+Milt^3^	1.08 (0.58, 2.00)	0.818
Milt+PM^4^	0.08 (0.02, 0.35)	0.0007
**Age**		
2–12 years	2.07 (1.20, 3.59)	0.0096
>12 years	Referent	
**Reported length of illness**		
< = 8 weeks	4.28 (1.32, 13.88)	0.0154
>8 weeks	Referent	

^1^Excludes 13 patients with treatment interruption or default, one patient treated for PKDL 2 months after VL treatment and 64 patients lost to follow-up at 6 months

^2^Single dose AmBisome

^3^AmBisome + miltefosine

^4^Miltefosine + paromomycin

### Safety analysis

Serious adverse events were infrequent in all study arms. There were 5 serious adverse events (SAE) in the SDA arm. Anaphylactic reaction occurred during treatment in one patient and was related to the study drug AmBisome. There were four SAEs after completion of treatment and discharge from hospital: one asymptomatic atrial ectopic possibly related to AmBisome and three other SAEs that were unrelated to the study drugs: TB empyema, hospitalization due to dehydration and elevated creatinine, and lower respiratory tract infection. All SAEs resolved completely with no sequela.

The most common adverse events were gastrointestinal (nausea, vomiting, diarrhoea, abdominal pain) and back pain (Table 5). Adverse events leading to treatment interruption were rare (<1% for all three drug regimens).

**Table 5:** Adverse events by treatment arm, intention-to-treat population (N = 1761).

	SDA^1^ (N = 891)	AmB+Milt^2^ (N = 358)	Milt+PM^3^ (N = 512)
Adverse events	n (%)	n (%)	n (%)
At least one AE reported	134 (15.0)	91 (25.4)	92 (18.0)
AEs leading to treatment interruption	4 (0.4)	2 (0.6)	1 (0.2)
Hypersensitivity reaction	2 (0.2)	1 (0.3)	0 (0)
Dermatitis	1 (0.1)	0 (0)	0 (0)
Severe Vomiting	0 (0)	1 (0.3)	1 (0.2)
Severe Abdominal pain	1 (0.1)	0 (0)	0 (0)
Serious AE diagnosed after end of treatment			
Asymptomatic atrial ectopic	1 (0.1)	0 (0)	0 (0)
Serious AE judged unrelated to treatment			
TB empyema	1 (0.1)	0 (0)	0 (0)
Dehydration and elevated creatinine	1 (0.1)	0 (0)	0 (0)
Lower respiratory tract infection	1 (0.1)	0 (0)	0 (0)
			
**Non-serious AEs**			
Abdominal pain or dyspepsia	13 (1.5)	20 (5.6)	19 (3.7)
Vomiting	43 (4.8)	61 (17.0)	45 (8.8)
Injection site pain or swelling	0 (0)	1 (0.3)	15 (2.9)
Back pain	42 (4.7)	9 (2.5)	0 (0)
Cough	14 (1.6)	9 (2.5)	4 (0.8)

^1^Single dose AmBisome

^2^AmBisome + miltefosine

^3^Miltefosine + paromomycin

## Discussion

Previous phase-3 randomized controlled trials have shown these regimens were non-inferior to treatment with standard amphotericin B deoxycholate with ITT final cure of 93·0% for SDA (95% CI 87.5–96.3), AmB+Milt 97·5% (95% CI 93.3–99.2), and Milt+PM 98·7% (95% CI 95.1–99.8) [12]. Cure rates by ITT in this study, while not quite as high, still achieved acceptable levels with the differences largely due to loss to follow-up. Earlier DND*i* conducted a phase-3 clinical trial in Bangladesh to assess safety and efficacy of short course combination regimens in field conditions at Upazila level that provided excellent efficacy outcome (≥95%) and very good safety profile [19].

The demographic characteristics of the population enrolled in the study correspond to those defined for this area, roughly 70% patients older than 12 years, 30% of them women of child-bearing age. The effectiveness in complete case analysis in this study was slightly higher in the Milt+PM arm than in the other two arms, which may partly be due to the higher number of clinically unwell patients being allocated to the SDA treatment arm.

Despite a range of clinical severity, presentations, and patient demographics, all of the treatments showed excellent safety profiles. This study was non-comparative, both SDA and combination of Milt+PM had satisfactory effectiveness of >90% which corroborates the decision by the Indian control program to use these treatments in the elimination program. No complications were seen in pregnant (treated with SDA) or extremely young patients. Generally, the treatments were easily prepared and administered by health care providers, and appeared to be well accepted by patients.

When elimination was first envisioned, oral miltefosine was proposed to be used primarily in the attack phase due to its acceptability. In parallel to the provisional results of this study, the WHO included India in the AmBisome donation programme, resulting in India adopting these new treatment modalities within the national elimination programme, replacing miltefosine monotherapy. To date, this has proved to be a very effective strategy, with over 12,000 patients having been treated in the attack phase with SDA within the public health sector across the Indian subcontinent with excellent safety and efficacy [14]. Although widely implemented, SDA is not without its limitations–complex storage and preparatory requirements mean that its safe use is contingent on logistical support that is not required for Milt+PM, for example. The unintended consequence of this has been the neglect of alternative drug combinations, which has resulted in a lack of stock and awareness of these regimens in the national programme.

Considering the limited number of therapeutic options available, it is critical to ensure that procurement and availability of all three drugs is ensured within the elimination framework. Currently, all three WHO supported formulations of these drugs are produced by single source manufacturers AmBisome (Gilead Sci., USA), miltefosine (Knight Therapeutic Inc., Canada), and paromomycin sulphate (Gland Pharma, India) [16], making the supply chain sensitive to factory and quality issues should they arise.

This reflects the urgent need for investment in bio-equivalence studies, technology transfer, and alternative production, which may potentially need to be centralized and pooled to ensure adequate market conditions. Moreover, all these limitations justify strengthening the development of new chemical entities (NCEs) that are needed in the form of short-course oral combinations, to replace the existing drugs in the Indian subcontinent and worldwide [20].

Although resistance to amphotericin B has yet to be demonstrated *in vivo* despite decades of use, prolonged use of monotherapies such as miltefosine and paromomycin have resulted in reduced drug susceptibility, and potential mechanisms of amphotericin B resistance have been described [21]. Reduced drug susceptibility for SSG and Milt were only determined well after they had progressed to unacceptable levels; as such it is critical that the national programme develops sentinel surveillance for drug susceptibility monitoring of VL drugs so that early signals can be generated that can guide more rational use of existing therapeutic options. Such initiatives are underway in India [22] but are yet to be developed in Bangladesh or Nepal. There are also a number of challenges that need to be considered for the Milt+PM and AmB+Milt regimens. Although compliance was very high in this study (>99%), this was based on patients being actively traced to complete treatment and a large proportion being managed as inpatients for the duration of treatment. The PHC system in Bihar remains weak and overburdened with long waiting times and irregular timings–thus returning daily for treatment for a period of 10 days becomes an additional economic burden for patients and caregivers and is likely to result in reduced treatment compliance. Additionally, for Milt containing regimens, there is a requirement for women of reproductive age to take a pregnancy test, and, if negative, to comply with contraceptive cover during treatment and for 3 months afterwards, something that has generally been poorly followed under programmatic conditions. Given that the most common adverse event related to Milt is vomiting, contraceptive injections remain the most suitable option, recently been made available in India within the public health sector [23]. As such, clear coordination and preparation on safety messaging for all treatments evaluated in this study is required.

There are a number of limitations to this study. Although it was originally planned that each site would use a particular regimen, there was a degree of mixing of treatments between sites. Additionally, children were under-represented due to regulatory demands, while the majority of patients receiving the Milt+PM arm received treatment as in-patients, reducing the validity of the feasibility interpretation of this arm in normative settings. Finally, the majority of patients in two of the treatment arms were treated by MSF doctors, supporting activities at Hajipur hospital.

This is the largest prospective study conducted using the revised WHO recommended VL treatment regimens for the Indian subcontinent, and to the authors’ knowledge, the first NTD based phase 4 study within the Indian subcontinent. The results were used by the Indian national programme to support policy change, introducing SDA and the different combinations as treatment options in the elimination strategy.

## Supporting information

S1 Table Allocation of drug regimens and baseline characteristics by recruitment site. (DOC)

S2 Table Characteristics of patients with and without 6m follow-up data. (DOC)

## References

[1] World Health Organization. Weekly Epidemiological Record: Leishmaniasis in high-burden countries: an epidemiological update based in data reported in 2014. P287. Accessible at http://www.who.int/wer/2016/wer9122.pdf?ua=1. Accessed August 20, 2017.

[2] Alvar J, Vélez ID, Bern C, Herrero M, Desjeux P, Cano J, et al Leishmaniasis worldwide and global estimates of its incidence. PLoS One. 2012;7(5). 10.1371/journal.pone.0035671 PMC3365071 22693548

[3] Programme NVBDCP. Kala-azar Cases and Deaths in the Country since 2010 [Internet]. 2017 [cited 2017 August 20]. Available from: http://nvbdcp.gov.in/ka-cd.html

[4] Berman J. Amphotericin B Formulations and Other Drugs for Visceral Leishmaniasis. Am J Trop Med Hyg [Internet]. 2014;92(3):471–3. Available from: http://www.ajtmh.org/cgi/doi/10.4269/ajtmh.14-0743 25510726 10.4269/ajtmh.14-0743 PMC4350529

[5] Sundar S, Chakravarty J. An update on pharmacotherapy for leishmaniasis. Expert Opin Pharmacother [Internet]. Informa UK, Ltd.; 2014;16(01):1–16. Available from: 10.1517/14656566.2015.973850 25346016 PMC4293334

[6] Jha TK. Drug unresponsiveness & combination therapy for kala-azar. Indian J Med Res. 2006;123(March):389–98. 16778318

[7] Ostyn B, Hasker E, Dorlo TPC, Rijal S, Sundar S, Dujardin JC, et al Failure of miltefosine treatment for visceral leishmaniasis in children and men in South-East Asia. PLoS One. 2014;9(6). 10.1371/journal.pone.0100220 PMC4062493 24941345

[8] Sundar Shyam, Singh Anup, Rai Madhukar, Prajapati Vijay K., Singh Avinash K., Ostyn Bart, Boelaert Marleen, Dujardin Jean-Claude, Jaya Chakravarty; Efficacy of Miltefosine in the Treatment of Visceral Leishmaniasis in India After a Decade of Use. Clin Infect Dis 2012; 55 (4): 543–550. 10.1093/cid/cis474 22573856

[9] Dorlo TP, Rijal S, Ostyn B, de Vries PJ, Singh R, Bhattarai N, Uranw S, Dujardin JC, Boelaert M, Beijnen JH, Huitema AD. Failure of miltefosine in visceral leishmaniasis is associated with low drug exposure. J Infect Dis. 2014 7 1;210(1):146–53) 10.1093/infdis/jiu039 24443541

[10] Rijal Suman, Ostyn Bart, Uranw Surendra, Rai Keshav, Narayan Raj Bhattarai Thomas P. C. Dorlo, Beijnen Jos H., Vanaerschot Manu, Decuypere Saskia, Dhakal Subodh S., Murari Lal Das Prahlad Karki, Singh Rupa, Boelaert Marleen, Jean-Claude Dujardin; Increasing Failure of Miltefosine in the Treatment of Kala-azar in Nepal and the Potential Role of Parasite Drug Resistance, Reinfection, or Noncompliance, *Clinical Infectious Diseases*, Volume 56, Issue 11, 1 6 2013, Pages 1530–1538, 10.1093/cid/cit102 23425958

[11] Sundar S, Chakravarty J, Agarwal D, Rai M, Murray HW. Single-dose liposomal amphotericin B for visceral leishmaniasis in India. N Engl J Med. 2010;362:504–12. 10.1056/NEJMoa0903627 20147716

[12] Sundar S, Sinha PK, Rai M, Verma DK, Nawin K, Alam S, et al Comparison of short-course multidrug treatment with standard therapy for visceral leishmaniasis in India: An open-label, non-inferiority, randomised controlled trial. Lancet [Internet]. Elsevier Ltd; 2011;377(9764):477–86. Available from: 10.1016/S0140-6736(10)62050-8 21255828

[13] World Health Organization. Control of the leishmaniases. Report of a meeting of the WHO Expert Committee on the Control of Leishmaniasis, Geneva, 22–26 March 2010 [Internet]. World Health Organization technical report series Geneva; 2010 1 p. 1–186.

[14] Van Griensven J, Boelaert M. Combination therapy for visceral leishmaniasis. Int J Infect Microbiol; 2013;2(9764):443–4. Available from: 10.1016/S0140-6736(10)62237-4 21255829

[15] Banjara M. Combination therapy for visceral leishmaniasis. Lancet. 2011;377(2):443–4. 21255829 10.1016/S0140-6736(10)62237-4

[16] Van Griensven J, Balasegaram M, Meheus F, Alvar J, Lynen L, Boelaert M. Combination therapy for visceral leishmaniasis. Lancet Infect Dis [Internet]. Elsevier Ltd; 2010;10(3):184–94. Available from: 10.1016/S1473-3099(10)70011-6 20185097

[17] http://www.who.int/childgrowth/software/en/ and http://www.who.int/growthref/tools/en/.

[18] http://www.who.int/vmnis/indicators/haemoglobin.pdf.

[19] Rahman R, Goyal V, Haque R, et al Safety and efficacy of short course combination regimens with AmBisome, miltefosine and paromomycin for the treatment of visceral leishmaniasis (VL) in Bangladesh. PLoS Negl Trop Dis. 2017;11(5):e0005635 10.1371/journal.pntd.0005635 28558062 PMC5466346

[20] Charles E. Mowbray. Anti-leishmanial Drug Discovery: Past, Present and Perspectives In: Drug Discovery for Leishmaniasis. (edits. Rivas L. & Gil C.). The Royal Society of Chemistry, 218 (402 pp.)

[21] Purkait B, Kumar A, Nandi N, et al Mechanism of Amphotericin B Resistance in Clinical Isolates of Leishmania donovani. *Antimicrobial Agents and Chemotherapy*. 2012;56(2):1031–1041. 10.1128/AAC.00030-11 22123699 PMC3264217

[22] WHO Library Cataloguing-in-Publication Data Kala-Azar elimination programme: report of a WHO consultation of partners, Geneva, Switzerland, 10–11 February 2015.

[23] https://timesofindia.indiatimes.com/india/NGOs-welcome-govts-move-to-introduce-injectable-contraceptive/articleshow/49104513.cms.

