# Supplementary material for: Field safety and effectiveness of new visceral leishmaniasis treatment regimens within public health facilities in Bihar, India
Source: PLoS Negl Trop Dis. 2018 Oct 22;12(10):e0006830. doi: 10.1371/journal.pntd.0006830 (PMC6197645; doi:10.1371/journal.pntd.0006830)
Supplement: S1 Table — (DOC) [file pntd.0006830.s001.doc]

**S1 Table: Allocation of drug regimens and baseline characteristics by recruitment site**

|  | **Chapra District Hospital (Saran)** | **Hajipur District Hospital (Vaishali)** | **RMRI** | **Saran PHCs** | **Vaishali PHCs** | **Total** |
| --- | --- | --- | --- | --- | --- | --- |
| **Regimen (ITT)** | **N=378** | **N=1052** | **N=96** | **N=120** | **N=115** | **N=1761** |
| SDA | 4 | 828 | 59 | 0 | 0 | 891 |
| AmB-Milt | 0 | 218 | 25 | 0 | 115 | 358 |
| Milt-PM | 374 | 6 | 12 | 120 | 0 | 512 |
|  |  |  |  |  |  |  |
| **Demographics** |  |  |  |  |  |  |
| % male | 56.4% | 60.2% | 69.8% | 69.2% | 62.6% | 1068 (61%) |
| Age <=12 (%) | 50.5% | 32.5% | 1.0% | 0.0% | 0.0% | 534 (30.3%) |
|  |  |  |  |  |  |  |
| **Clinical characteristics** |  |  |  |  |  |  |
| Weeks of illness |  |  |  |  |  |  |
| Median [IQR] | 4 [4,8] | 4 [3,8] | 8 [4,15] | 4 [3,8] | 4 [4,8] |  |
| Range | 0 - 48 | 1 - 52 | 1.5 – 144 | 1.5 - 48 | 1 - 48 |  |
| Severe anemia (%) | 71 (18.9) | 389 (37.0) | 54 (56.3) | 33 (27.5) | 31 (27.0) | 578 (32.8%) |
| Severe wasting (%) | 67 (17.5) | 167 (15.9) | 8 (8.3) | 19 (15.8) | 9 (7.8) | 270 (15.3%) |
| ALT >=200 (%) | 5 (1.3) | 46 (4.4) | 1 (1.0) | 6 (5.0) | 2 (1.7) | 60 (3.4%) |
| AST >=200 (%) | 21 (5.6) | 123 (11.7) | 3 (3.1) | 11 (9.2) | 9 (7.8) | 167 (9.5%) |
