# Supplementary material for: Field safety and effectiveness of new visceral leishmaniasis treatment regimens within public health facilities in Bihar, India
Source: PLoS Negl Trop Dis. 2018 Oct 22;12(10):e0006830. doi: 10.1371/journal.pntd.0006830 (PMC6197645; doi:10.1371/journal.pntd.0006830)
Supplement: S2 Table — (DOC) [file pntd.0006830.s002.doc]

**S2 Table:** Characteristics of patients with and without 6m follow-up data

|  | **With 6 month outcome data N=1697** | **Lost to follow-up at 6 months N=64** | **p value** |
| --- | --- | --- | --- |
| **Drug regimen N (row %)** |  |  |  |
| Ambisome | 859 (96.4) | 32 (3.6) | 0.005 |
| AmB-Milt | 336 (93.9) | 22 (6.2) |  |
| Milt-PM | 502 (98.1) | 10 (2.0) |  |
| **Recruitment site N (row %)** |  |  |  |
| Chapra Sadar District Hospital | 371 (98.2) | 7 (1.9) | <.0001 |
| Hajipur Sadar District Hospital | 1021 (97.1) | 31 (3.0) |  |
| RMRI | 86 (89.6) | 10 (10.4) |  |
| Saran district PHCs: | 119 (99.2) | 1 (0.8) |  |
| Vaishali PHCs: | 100 (87.0) | 15 (13.0) |  |
| **Demographic characteristics** |  |  |  |
| Median age (years [IQR]) | 20 [10, 35] | 33.5 [20, 50] | <.0001 |
| Age range (years) | 2 - 80 | 3.5 - 63 |  |
| Age < 12 years N (row %) | 528 (98.9) | 6 (1.1) | 0.0002 |
| Age > 12 years N (row %) | 1169 (95.3) | 58 (4.7) |  |
| Male N (row %) | 1023 (95.8) | 45 (4.2) | 0.11 |
| Female N (row %) | 674 (97.3) | 19 (2.7) |  |
|  |  |  |  |
| **Clinical characteristics** |  |  |  |
| Weeks of illness |  |  |  |
| Median [IQR] | 4 [3, 8] | 6 [3.5, 12] | 0.17 |
| > 8 weeks | 356 (21.0) | 18 (28.1) | 0.17 |
| Severe wasting N (column %) | 264 (15.6) | 6 (9.4) | 0.22 |
| Hemoglobin (mean [SD] in g/L) | 8.9 (2.0) | 8.8 (2.3) | 0.56 |
| Severe anemia (column %) | 551 (32.5) | 27 (42.2) | 0.1 |
| Creatinine (mean [SD] in μmol/L) | 0.74 (0.27) | 0.87 (0.27) | <.0001 |
| Alanine aminotransferase |  |  |  |
| Median[IQR] | 33.6 [19.0,63.5] | 37.3 [24.4,55.6] | 0.76 |
| Marked elevation (>=200) N(%) | 60 (3.5) | 0 (0) |  |
| Aspartate aminotransferase |  |  |  |
| Median[IQR] | 56.3 [36.6,98.6] | 57.4 [40.9,74.8] | 0.63 |
| Marked elevation (>=200) N(%) | 162 (9.6) | 5 (7.8) |  |
